# *Bos taurus–indicus* hybridization correlates with intralocus sexual-conflict effects of *PRDM9* on male and female fertility in Holstein cattle

**DOI:** 10.1186/s12863-019-0773-5

**Published:** 2019-08-28

**Authors:** Eyal Seroussi, Andrey Shirak, Moran Gershoni, Ephraim Ezra, Daniel Jordan de Abreu Santos, Li Ma, George E. Liu

**Affiliations:** 1 Agricultural Research Organization (ARO), Volcani Center, Institute of Animal Science, HaMaccabim Road, P.O.B 15159, 7528809 Rishon LeTsiyon, Israel; 2 Israel Cattle Breeders Association, Caesarea, Israel; 3 Department of Animal and Avian Sciences, University of Maryland, College Park, MD USA; 4 Animal Genomics and Improvement Laboratory, BARC, Agricultural Research Service, USDA, Beltsville, MD 20705 USA

**Keywords:** Genomic conflict, Recombination, Dairy, Beef, Fertility, Holstein

## Abstract

**Background:**

Crossover localization during meiotic recombination is mediated by the fast-evolving zinc-finger (ZnF) domain of gene *PRDM9*. To study its impact on dairy cattle performance, we compared its genetic variation between the relatively small Israeli (IL) Holsteins and the North American (US) Holsteins that count millions.

**Results:**

Initially, we analyzed the major BTA1 haplotypes present in IL Holsteins based on the 10 most telomeric SNPs of the BovineSNP50 BeadChip. Sequencing of representative haplotype carriers indicated that for all frequent haplotypes (> 6%), the variable *PRDM9* ZnF array consisted of seven tandem ZnF repeats. Two rare haplotypes (frequency < 4%) carried an indicine *PRDM9*, whereas all others were variants of the taurine type. These two haplotypes included the minor SNP allele, which was perfectly linked with a previously described *PRDM9* allele known to induce unique localization of recombination hotspots. One of them had a significant (*p* = 0.03) negative effect on IL sire fertility. This haplotype combined the rare minor alleles of the only SNPs with significant (*p* < 0.05) negative substitution effects on US sire fertility (SCR). Analysis of telomeric SNPs indicated general agreement of allele frequencies (*R* = 0.95) and of the substitution effects on sire fertility (SCR, *R* = 0.6) between the US and IL samples. Surprisingly, the alleles that had a negative impact on male fertility had the most positive substitution effects on female fertility traits (DPR, CCR and HCR).

**Conclusions:**

A negative genetic correlation between male and female fertility is encoded within the BTA1 telomere. Cloning the taurine *PRDM9* gene, which is the common form carried by Holsteins, we encountered the infiltration of an indicine *PRDM9* variant into this population. During meiosis, in heterozygous males, the indicine *PRDM9* variant may induce incompatibility of recombination hotspots and male infertility. However, this variant is associated with favorable female fertility, which would explain its survival and the general negative correlation (*R* = − 0.3) observed between male and female fertility in US Holsteins. Further research is needed to explain the mechanism underlying this positive effect and to devise a methodology to unlink it from the negative effect on male fertility during breeding.

**Electronic supplementary material:**

The online version of this article (10.1186/s12863-019-0773-5) contains supplementary material, which is available to authorized users.

## Background

During meiosis, genetic recombination reshuffles homologous chromosomes to produce offspring with combinations of traits that differ from those of their parents. Thus, increased recombination rate is considered to be essential for effective selection during domestication [1, 2], and this trait has recently drawn much attention from cattle researchers and breeders [3–8].

Among others, the genes REC8 Meiotic Recombination Protein (*REC8*)*,* Ring Finger Protein 212 (*RNF212*) and Cyclin B1 Interacting Protein 1 (*CCNB1IP1*) have been implicated in driving variation in meiotic recombination rate, with PR/SET Domain 9 (*PRDM9*) controlling the positioning of recombination hotspots in ruminants, as in other mammals [3, 5, 6, 9]. *PRDM9* is annotated at the telomeric end of *Bos taurus* autosome 1 (BTA1) (GenBank: NP_001306826) including four major functional domains, two of which, Krüppel Associated Box (KRAB) and SSX Repression Domain (SSXRD) nuclear localization signal, are associated with transcription repression. This transcription-repression-like module is followed by a SET domain that provides methyltransferase activity and a C2H2 zinc finger (ZnF) array that binds to DNA. During meiosis, the ZnF array directs the specific binding of PRDM9 to sites across the chromosomes, and the SET domain produces H3K4me3 and H3K36me3 trimethylations to nearby histones [10]. These modifications serve to recruit the SPO11 initiator of meiotic double stranded breaks topoisomerase (SPO11) to initiate double-strand breaks by a mechanism that involves protein–protein interactions with PRDM9’s transcription-repression-like module and that eventually promotes crossing over [10].

The C2H2 ZnF array of *PRDM9* is the fastest evolving ZnF in humans and other mammals [11]; this is compatible with the evident selection at the DNA-binding sites of PRDM9 [12]. This variation may promote subfertility and male sterility in hybrids, in which *PRDM9* plays a complex role (reviewed by [10]). In the dairy sector, subfertility accounts for major economic losses, and dairy cattle breeding, which focuses primarily on selection for production traits, has resulted in a decline in the reproductive performance of Holstein cows [13]. Fertility issues are also predominant in male crossbreds of *Bos taurus* × *B. indicus* cattle. Compared to purebreds, crossbred progeny of Holstein–Friesian and indicine cattle show poorer seminal parameters, subfertility and male sterility [14]. The current bovine *PRDM9* reference sequence originates from beef cattle (US Hereford cattle) and despite much interest in this gene’s function in dairy cattle, there is no Holstein *PRDM9* reference sequence deposited in GenBank. In this study, we describe a longer form of PRDM9 protein that is prevalent in Holstein cattle and analyze the effects of the different forms on male and female fertility.

## Results

### Computerized cloning of PRDM9 of an influential Israeli (IL) Holstein sire

**Fig. 1:**
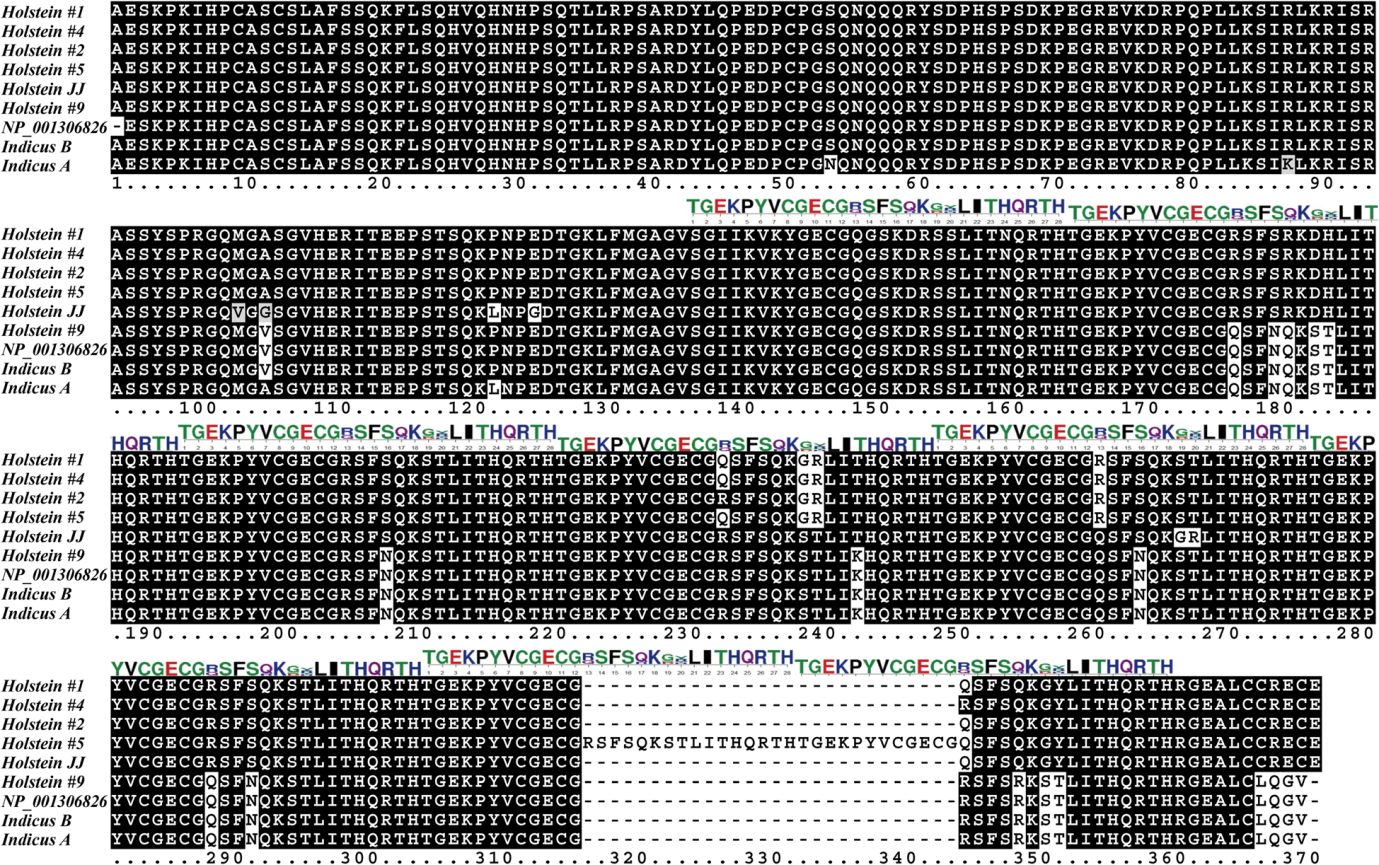
The putative amino-acid variation encoded in exon 10 of *PRDM9*. Genomic DNA of sires preferentially homozygous for haplotype alleles were used as templates for amplification and for Sanger sequencing using nucleotide primers 1 and 2 in Additional file 1: Table S1. The resulting traces were compared to reference sequences (*Bos taurus* [GenBank: NP_001306826.2] and two variants of *B. indicus* based on sequence phasing [GenBank: XP_019820291.1 and ANN45578]) or assembled sequences from NGS data (Holstein, JJ [ENA: LR536713]). The amino-acid alignment was colored using Boxshade. Dashes indicate gaps introduced by the alignment program or stop codons. Identical and similar amino acid residues in at least two of four sequences are indicated by a black and gray background, respectively. White boxes indicate non-conservative amino acid changes between the proteins. Above the alignment, tandem ZnF repeats are labeled following Zhou et al.*,* 2018 [15]. Each repeat consists of 28 residues. Nomenclature of variants follows that of their associated BTA1 telomeric SNP haplotypes (Table 3) and theirs DNA encoding sequences (Additional file 1)

To obtain the *PRDM9* sequence of a representative Holstein sire (JJ, HOLISRM000000007424), we applied deep sequencing to the genome of this leading Israeli service sire. At the end of 2018, this sire was recorded in the top 20 sires for total net merit, with more than 10,000 daughters. Being a descendant of the popular US bull O-Bee Manfred Justice (HOUSA000122358313), this sire represents an influential blood line of Holstein cattle. Directed assembly resulted in a 13,568-bp gene (starting the count in the 5′ untranslated region, Table 1) covered by 2147 reads of 100 bp each (~ 16-fold coverage). As the assembly algorithm setup required a minimal match of 98 bp, all reads were of high quality, with no variation, mismatches or gaps (see BAM-format file [ENA: ERR3237582]). This assembled sequence had 99% nucleotide sequence identity with the reference mRNA sequence of *PRDM9* (GenBank: NP_001306826.2) and similarly, consisted of 10 exons all bordered by canonical splice sites (Table 1). The first nine exons were capable of encoding 383 amino acids that were all identical to those of the reference gene and comprised the transcription-repression-like module, followed by the SET domain. The last exon was capable of encoding 344 amino acids, which showed only 93% identity to their counterparts in the reference protein (Fig. 1). Hence, the fast-evolving ZnF array encoded by this exon was the source of all variation between the dairy and beef forms of *PRDM9,* resulting in a longer 727-amino-acid dairy variant compared to the reference protein of 725 amino acids from beef cattle (Fig. 1).

**Table 1:** Genomic organization of the *Bos taurus PRDM9* gene (using the representative Holstein sire)

Intron^a^	Exon	Intron		
	no.	size		size
TGAGCACTCCA ***ATG*** GCC	1	75	CCCACG **gt** gagaggca	406
attttcctt **ag** GCCAAA	2	124	CTATAG **gt** aacaggaa	307
cttctttcc **ag** GTTTCA	3	108	AGCAAG **gt** gaggggcc	4190
tcatttttt **ag** GTAAAC	4	50	CAGAAT **gt** gagtattt	1784
tcatgtgga **ag** ACAATG	5	157	AAGTCG **gt** aagagaaa	842
ctccatctt **ag** AACTCA	6	102	ACCTCT **gt** gagtgccc	216
ctcacctcc **ag** ATTGTG	7	272	TGGCTG **gt** gagaaaca	576
ctgacactc **ag** ATCACC	8	68	GATGAG **gt** gagtgcag	1145
ctcgacccc **ag** GTATGT	9	194	CCAGAG **gt** gagcgcca	1677
tcctctttc **ag** CAGAAT	10	1294	GAATCATAGccaacaaactacattttagtcacaggagaattactgcagccaccccatgcctcagctctaagggggcctcagaggaggtctgtgacctgttacagtcaccaagagtgtgaggagagacttcccaggtggtccaggggccaagactccaattcagggacccatgttcaatacctgattggggaactagctcccacatgctgcaactaagacacagtgcagctgAATAAAtaaatacgtaaataaatattttaaa...+poly-A+tail?	

^a^ Exon and intron sizes are given in base pairs. Intron and exon sequences are written in lowercase and uppercase letters, respectively. The first and last two bases of the introns (**gt** and **ag** for donor and acceptor splice sites, respectively) are in bold type. The initiation and stop codons, and the putative polyadenylation signal (***ATG***, ***TAG***, ***AATAA***) are in bold and underlined type. Starting from the initiation codon, the genomic and transcript sizes of the *PRDM9* gene as presented here were 13,557 and 2444 bp, respectively

### *PRDM9* expression

Length differences between the 727-amino-acid Holstein *PRDM9* variant and the 725-amino-acid reference sequence were also introduced by a different splicing scheme for the last exon. As indicated in Table 1, in our build, the splice donor is the first CAG motif 5′ of this exon, which is followed by another identical motif used by the reference. To verify which is the actual splicing donor, we explored RNA-Seq data deposited in the Sequence Read Archive (SRA) of NCBI. Expression was detected only in the testis and using a stringent SRA BLAST search, we located 414 reads from three RNA-Seq submissions of the Hereford SuperBull 99,375 testis (Domino). Of these reads, 265 were assembled into a 2586-bp complete cDNA (Fig. 1; [ENA: ERR3237910] for BAM-format and [ENA: LR536714] for annotated transcript sequence), which validated the first CAG motif as the donor (also exemplified by [SRA: SRR5363137.1086298]).

### Paralogous genes

**Fig. 2:**
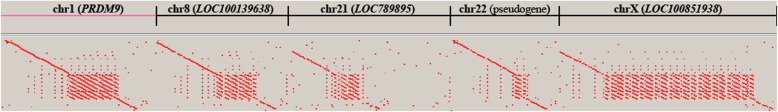
*PRDM9* ZnF array paralogs. Genomic reads of US Holsteins with sequence similarity to exon 10 of *PRDM9* were downloaded from the SRA database and assembled using GAP5 software. Each red dot represents an 8-bp repeat that is similar to the *PRDM9* exon 10 sequence. The domain of the tandem repeats forms a dotted rectangle, which reflects the number of tandem repeats

To further analyze variation in the Holstein *PRDM9* gene, it was essential to investigate and map close paralogous sequences that may interfere with this gene’s characterization. Using as template query the 13,598-bp sequence of the dairy form of *PRDM9*, we BLAST searched the current genome build (ARS-UCD1.2). This indicated the existence of five close paralogs (maximal score > 2500): *PRDM9* on BTA1 (identity 99%, coverage 100%), *LOC100851938* on BTAX (identity 89%, coverage 99%), *LOC100139638* on BTA8 (identity 92%, coverage 99%), *LOC789895* on BTA21 (identity 82%, coverage 95%), an unannotated *PRDM9*-like pseudogene on BTA22 (identity 92%, coverage 40%) (Fig. 2). It should be noted that the latter is annotated as *LOC113880961* in the hybrid cattle genome but not in the *B. taurus* genome build.

Diversity of the Holstein *PRDM9*’s ZnF array was further characterized by de-novo assembly of all Holstein reads in the SRA that have been deposited by the USDA (12 bulls, [NCBI BioProject: PRJNA277147]) and that were proved similar to the reference sequence of *PRDM9* exon 10 by an SRA BLAST search. This assembly resulted in five major contigs with varying lengths of ZnF arrays, ranging from 4 ZnF repeats on BTA22 to over 20 ZnF repeats on BTX, and corresponding to the five aforedescribed paralogs (Fig. 2). All reads that were assembled into the *PRDM9* contig matched its dairy form of seven repeats. We used this information to design PCR primers (Additional file 1: Table S1) that would enable specific amplification, and to apply Sanger sequencing of the major variation in the ZnF array of Holstein *PRDM9*.

### Haplotype analysis

Using PLINK software [16] sliding-window analysis over BTA1, we identified informative haplotypes of 10 single-nucleotide polymorphisms (SNPs) spanning the *PRDM9* locus (Table 2). Scores for the male fertility trait were calculated using a linear sire model that included the insemination technician as random effect and were based on determination of pregnancy by veterinary examination for all cows that did not display estrus within 60 d of insemination [17]. Input data included genotypes of 1750 sires for 10 polymorphic SNPs on BTA1 that fit the Hardy–Weinberg distribution (*p* < 0.001). The PLINK permutation option was employed to verify probability of association of the haplotype alleles with male fertility. The identified haplotype consisted of the most telomeric SNPs on the BovineSNP50K BeadChip at positions 157,229,645–157,542,408 (build ARS-UCD1.2), nearest to the *PRDM9* gene (157,545,780–157,559,387). For this window, 16 common haplotypes explained > 92% of the observed sequence variation (Table 3). The likelihood of association with male fertility was significant only for haplotype #9, which associated with negative male fertility (β value − 0.58, Table 3). This haplotype combined the rare minor alleles of the two SNPs that displayed the most negative effects on this trait (allele frequencies 3.7 and 9.1% with β values of − 0.5 and − 0.3, respectively, Table 2). However, since this simplified analysis may be confounded by population stratification, we applied bootstrapping with 100,000 permutations, which corroborated the significance of this association (Table 3).

**Table 2:** BTA1 telomeric SNPs

SNP marker^a^	BTA1 position	A1 (minor)	A2	MAF^b^	β^c^
*ARS-BFGL-NGS-73542*	157,229,645	A	G	0.4020	−0.01
*ARS-BFGL-NGS-19721*	157,253,652	G	A	0.4090	0.04
*ARS-BFGL-NGS-101788*	157,307,208	A	G	0.3740	−0.02
*BTA-105868-no-rs*	157,328,448	A	G	0.2400	0.10
*BTB-01585499*	157,367,221	G	A	0.2910	−0.08
ARS-BFGL-NGS-113905	157,405,441	A	G	**0.0373**	**−0.50**
*ARS-BFGL-NGS-90894*	157,431,081	A	G	0.1570	0.04
ARS-BFGL-NGS-83544	157,458,860	A	G	**0.0913**	**−0.30**
*Hapmap26498-BTA-33060*	157,503,718	A	G	0.3490	−0.05
*ARS-BFGL-NGS-76717*	157,542,408	G	A	0.2350	−0.16

^a^ Boldfaced names indicate SNP alleles with exceptionally low frequency and β values

^b^ Frequency of the minor allele was calculated based on 1750 BeadChips

^c^ Marker effects on male fertility were estimated using PLINK Fisher’s exact test

**Table 3:** Association analysis of BTA1 telomeric SNP haplotypes with male fertility

Haplotype^a^	#	Freq^b^	β^c^	STAT	*p*	EMP1^d^
*GAGGAGGGGA*	1	0.2760	0.0749	0.517	0.472	0.4718
*AGAGGGGGAG*	2	0.1190	−0.1120	0.670	0.413	0.4133
*AGAAAGGGGA*	3	0.0981	0.1570	0.969	0.325	0.3266
*AAGGGGAGGA*	4	0.0802	0.2560	2.100	0.148	0.1493
*GGAAAGAGGA*	5	0.0533	−0.0120	0.004	0.952	0.9518
*GAGGAGGGAG*	6	0.0530	−0.2140	1.050	0.306	0.3071
*GGAAAGGGAA*	7	0.0504	0.3230	2.070	0.151	0.1498
*GAGGAGGGAA*	8	0.0458	0.0161	0.005	0.945	0.9438
GAGGAAGAGA	**9**	**0.0370**	**−0.5800**	**4.560**	**0.033**	**0.0325**
*GAGGGGGAGA*	10	0.0257	0.1530	0.263	0.608	0.6043
*GGGGAGGGAG*	11	0.0184	0.5370	2.590	0.108	0.1073
*AAGGAGGGAG*	12	0.0154	−0.0296	0.006	0.937	0.9361
*AAGGAGGGGA*	13	0.0152	0.0878	0.037	0.847	0.8467
*AGGGAGGGGA*	14	0.0148	−0.2660	0.481	0.488	0.4859
*AGGAAGGGAG*	15	0.0124	−0.1210	0.090	0.764	0.7601
*AGAAGGGAGA*	16	0.0103	0.1830	0.131	0.717	0.7139

^a^ Boldfaced haplotype has a significantly low β value

^b^ Haplotype frequency was used to sort this table

^c^ Haplotype effects on male fertility were estimated using PLINK linear regression test

^d^ Empirical *p* value was the number of times the permuted haplotype-statistic exceeded *p* in 100,000 permutations

We further analyzed the selected haplotype using a large-scale pedigree haplotyper [18]; we examined the statistically phased haplotypes and adjusted their reconstruction based on Mendelian inheritance and the complex kinship relations within the sample. The final sample for which association of the *PRDM9* locus with the male fertility trait was estimated included 1414 sires with fully reconstructed and confirmed haplotypes. This analysis indicated that haplotype #9 is associated with sires with a negative score for male fertility (chi-squared test, *p* < 0.05, Table 3).

### Confirming PRDM9 association with fertility in US Holsteins

While the association analysis is somewhat limited when using the data for the IL Holstein herd, the US population offers almost unlimited statistical power as it includes millions of individuals with Illumina BeadChip data. We used this dataset to test the association between fertility traits and nine BTA1 telomeric SNPs that were genotyped in both the US and IL datasets (Table 4). For these SNPs, allelic composition was very similar (*R* = 0.95) to that observed in the IL population (Table 2). Table 4 shows that all effects were significant, most of them reaching the lowest number possible by the computing software for occurrence by chance, and thus their *p* values were indistinguishable from zero. We observed a significant correlation (0.6) between the substitution effects on the male fertility trait estimated by the sire conception rate (β SCR values, Table 4) and the effects of these SNPs on male fertility in the IL Holstein herd (β values, Table 2). This significant correlation indicates that the trends measured for the much smaller (2576-fold) IL substitution effects (Table 2) were also real. Indeed, for the US population as well, only the two SNPs with the lowest minor allele frequency (MAF < 10%, Table 4), which are carried by the aforedescribed *B. indicus* haplotype, had negative effects on sire fertility (Table 4). Surprisingly, these two SNPs were the only ones with positive substitution effects on female fertility represented by heifer conception rate (β HCR values, Table 4). As other traits of female fertility, including rates for daughter pregnancy (DPR) and cow conception (CCR), were positively correlated with HCR (Table 5), similar effect values were also observed for these other traits (data not shown). This suggests that near the BTA1 telomere, there is a linkage between a beneficial allele that affects female fertility and an allele that reduces male fertility. These observations were supported by the moderate negative genetic correlations (*R* ≈ − 0.3, on average) that were generally noticed between male (SCR) and female (DPR, HCR and CCR) fertility traits in the US sample (Table 5). Consequently, strong negative correlations were observed between the effects of the BTA1 telomeric SNPs on US HCR with either IL male fertility or US SCR (*R* = − 0.78 and − 0.89, respectively, Table 4).

**Table 4:** Substitution effects on fertility traits in US Holstein cattle of BTA1 telomeric SNPs

SNP marker^a^	MAF^b^	β HCR^c^	P_HCR_	β SCR^c^	P_SCR_
*ARS-BFGL-NGS-73542*	0.4158	−0.226	0	0.015	0
*ARS-BFGL-NGS-19721*	0.3315	−0.280	0	0.025	0
*ARS-BFGL-NGS-101788*	0.3295	−0.250	0	0.023	0
*BTA-105868-no-rs*	0.2140	−0.126	1.4E-205	0.004	5.39E-21
*BTB-01585499*	0.2289	−0.315	0	0.044	0
ARS-BFGL-NGS-113905	**0.0833**	**0.064**	**4.93E-26**	**−0.011**	**4.35E-56**
*ARS-BFGL-NGS-90894*	0.1095	−0.208	0	0.034	0
ARS-BFGL-NGS-83544	**0.0953**	**0.046**	**2.63E-15**	**−0.001**	**0.05**
*Hapmap26498-BTA-33060*	0.2591	−0.259	0	0.042	0
Correlation with IL values^d^	0.95	−0.78		0.60	

^a^ Boldfaced names indicate SNP alleles with exceptional frequency and β values

^b^ Minor alleles were the same as in Table 2 and their frequencies were calculated based on 4,508,642 BeadChips

^c^ Marker effects on heifer and sire conception rates (HCR and SCR, with numbers of observations of 922,893 and 903,690, respectively) were estimated using PLINK Fisher’s exact test

^d^ Correlations (R) are with Table 2 β values. R was − 0.89 within the β values of Table 4

**Table 5:** Pearson correlations between EBVs of rates of daughter pregnancy and of sire, heifer and cow conception in the US Holstein population^a^

	DPR	HCR	CCR
SCR	−0.280	−0.247	− 0.368
DPR		0.452	0.880
HCR			0.614

^a^ DPR, daughter pregnancy rate; SCR, HCR, CCR, sire, heifer and cow conception rates, respectively

### Sequence analysis of exon 10 of PRDM9 and its encoded ZnF array

The rapidly evolving ZnF array encoded by exon 10 is thought to confer sequence specificity to the binding of *PRDM9* to DNA sites in which recombination hotspots are induced. Thus, variation of this domain in heterozygotes may drive incompatibility that affects male fertility. To analyze such variation, we Sanger sequenced this ZnF array in a sample of individuals that were preferentially homozygous for the common haplotype alleles of the BTA1 telomeric end (haplotypes 1–10; Table 3, Fig. 1, and Additional file 1). Haplotypes #9 and #10 were sequenced from heterozygotes using allele-specific PCR primers (Additional file 1: Table S1) or by subcloning into a sequencing vector. Such plasmid sequencing also enabled the identification of a *PRDM9* variant with eight ZnF repeats, which was carried by the relatively rare haplotypes #5 and #7. The nucleotide sequence of this variant was virtually identical to that of the most common allele (haplotype #1) except for an insertion in an additional sequence motif of the ZnF repeat (Fig. 1, [ENA: LR536717]).

**Fig. 3:**
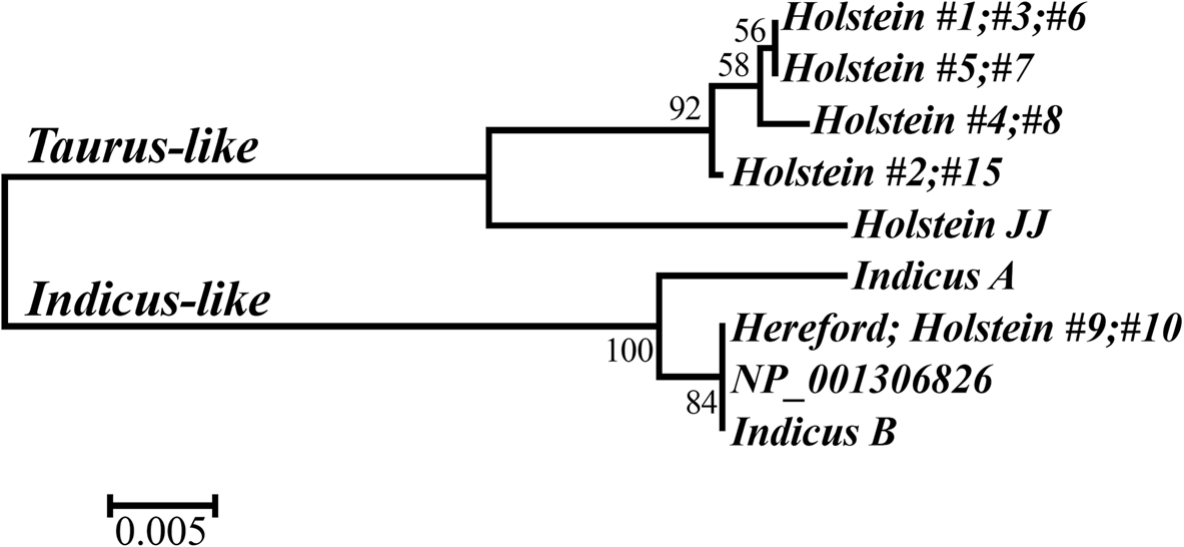
Phylogenetic tree of *PRDM9* ZnF array alleles. The evolutionary history of the polypeptides presented in Fig. 1 was inferred using the Neighbor-Joining method. The different alleles are identified by their carrying haplotype numbers. The optimal tree with the sum of branch length = 0.099 is shown. Next to the branches, the percentages of replicate trees in which the associated polypeptides clustered together in the bootstrap test are shown. The tree is drawn to the scale shown in units of number of amino acid substitutions per site

**Fig. 4:**
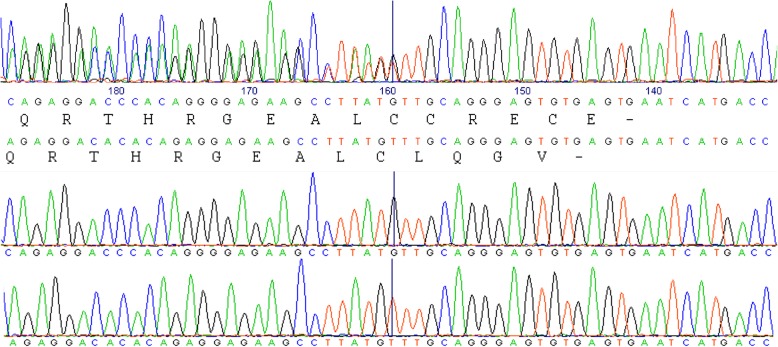
Ambiguous trace chromatograms associated with heterozygosity for the dairy and beef forms of *PRDM9*. On top, the ambiguous trace chromatogram was obtained by sequencing of the PCR product amplified from sire #5228 that carries haplotypes #9 and #15 (Table 3). Sequencing was performed using the reverse primer (primer 1, Additional file 1: Table S1). Phased nucleotides and their corresponding encoded protein translations are presented below this chromatogram. Further below, chromatograms were obtained from bacterial-cloned fragments amplified from sire #5611 that carries haplotypes #8 and #10 using the SP6 primer

Analysis of the variation of the *PRDM9* ZnF-array alleles indicated their division into two phylogenetic groups (Fig. 3). Most forms belonged to the longer 727-amino-acid dairy variant (Fig. 1), which we refer to as taurus-like type (Fig. 3). Individuals heterozygous for the rare haplotypes #9 and #10, which were the only haplotypes that carried the minor allele ‘A’ in the SNP marker ARS-BFGL-NGS-83544 (~ 9% of the population, Table 2), were characterized by ambiguous trace chromatograms when sequenced in the reverse orientation (Fig. 4). Such forms are compatible with the presence of the 725-amino-acid *PRDM9* variant, which we refer to as the indicus-like branch (Fig. 3). This shorter form was also present in the *B. taurus* reference sequence and in the sequence of Dominette as assembled from trace files (data not shown), both derived from the Hereford beef breed; and in the reference sequences for *B. indicus PRDM9*. Hence, haplotype #9, which was associated for sires with a negative score for male fertility, was also associated with the indicus-like *PRDM9*, suggesting that it drives male infertility as in *Bos taurus–indicus* hybrids.

## Discussion

The purpose of this study was to discover *PRDM9* alleles that affect dairy production traits or could lead to more rapid genomic selection in Holstein cattle breeding by controlling the rates of meiotic recombination. On the one hand, meiotic recombination driven by *PRDM9* may induce deleterious chromosomal instability and meiotic drive [11, 19, 20]; on the other, it reshuffles paternal and maternal genetic alleles in the next generation, potentially providing better novel combinations of genetic variants [15]. A recent study of US cattle showed that a specific *PRDM9* allele denoted ‘allele 5’ has a dramatic influence on the localization of recombination hotspots and unique recombination hotspot regions that are distinguishable from hotspot regions modulated by all other alleles [15]. However, it was unclear whether this pattern would also be observed in IL Holstein cattle, which have a different demographic history, although AI using semen of elite US sires is often practiced to enhance the local variety. We applied genomic sequencing as the putative method of choice to investigate which *PRDM9* alleles are prevalent in the IL Holsteins. Computerized cloning of the *PRDM9* gene of an influential IL Holstein sire indicated that it encodes a 727-amino-acid *PRDM9* variant, which we refer to as the dairy form. This form was longer than the beef form of the GenBank reference sequence, which was derived from the Hereford beef breed, as this breed’s genome was the first to be sequenced, assembled, and annotated [21]. Moreover, this reference suggests an alternative splicing which does not correspond to the common transcript, as evidenced from our assembly of RNA-Seq data obtained from Hereford SuperBull 99,375 testis; as such, it should be considered a computational artifact. Based on this RNA-Seq read assembly, we give the correct transcript sequence of the beef form.

As expected [19], most of the nucleotide variation was observed within the repetitive ZnF array. However, in both forms, we mostly observed seven tandem ZnF repeats, while other repeat numbers have also been previously suggested [15]. Taking into account paralogous sequences, we carefully assembled NGS data of 12 US bulls, and concluded that all observed *PRDM9* alleles have at least seven tandem ZnF repeats, while smaller repeat numbers belong to paralogous loci. To strengthen this conclusion, we analyzed the major BTA1 haplotypes present in IL Holsteins based on the 10 most telomeric SNPs available on the Illumina BovineSNP50 BeadChip. Sanger sequencing of representative haplotype carriers indicated that for all frequent haplotypes (frequency > 6%), the sequences of the *PRDM9* ZnF array consisted of seven tandem ZnF repeats. Nevertheless, two rare haplotypes (frequency < 4%, #9 and #10, Table 3) carried the beef form of *PRDM9*, while all others were variants of the dairy type. These two haplotypes included the minor SNP allele ‘A’ of rs110661033 or ARS-BFGL-NGS-83544 which was perfectly linked with allele 5 of *PRDM9* [15]. Hence, these are likely to induce different localization of the recombination hotspots compared to all other haplotype alleles, as has been previously reported [15]. Moreover, haplotype #9 had a significant (*p* = 0.03) negative effect on IL sire fertility. This haplotype combined the rare minor alleles of the only SNPs tending to negative substitution effects on IL sire fertility (Table 2). To ensure this observation’s significance, we analyzed nine of the most telomeric BTA1 SNPs using data from the US national dairy cattle database that includes records for millions of individuals (Table 4). This analysis indicated general agreement between the allele frequency (*R* = 0.95) and the substitution effects on sire fertility (SCR, *R* = 0.6) between the US and IL samples, confirming significant (*p* < 0.05) negative substitution effects on male fertility for both minor SNP alleles that associate with the IL #9 haplotype that carries the beef form of *PRDM9*. Surprisingly, the very same alleles had the most positive substitution effects on female fertility traits (DPR, CCR and HCR, exemplified for the latter in Table 4). This made us double-check our methodology, but realizing that “nothing in genetics makes sense except in light of genomic conflict” [22], we concluded that our results may point to a fundamental intralocus sexual conflict that arises for either the *PRDM9* gene or closely linked genes at the BTA1 telomere. Such situations in which a genetic locus combines beneficial alleles for females with a selective disadvantage for males have been frequently observed (recently reviewed, [23]). This may stabilize the survival of alleles with a negative impact on fertility despite the obvious importance of this trait to genetic fitness. Indeed, in the US Holstein population, we observed a moderate negative genetic correlation between male and female fertility traits (*R* ~ − 0.3, Table 5); this can now be explained by the intralocus sexual conflict on the BTA1 telomere, where we recorded a much higher negative genetic correlation (R ~ 0.9) between the substitution effects on female and male fertilities (Table 4). Such a moderate negative correlation between male and female fertility has been observed in Danish cattle, leading to the suggestion that in breeding schemes for fertility, attention should be focused on the female side [24]. As in IL, but unlike in the US [25], male semen is not titrated according to male fertility score; it may be that the IL breeding scheme led to a much lower frequency (< 4%, 2.2-fold less than in the US, Tables 2 and 4) of the minor SNP allele of ARS-BFGL-NGS-113905. This allele has the highest negative impact on male fertility and thus the selection in IL against this allele decreased the negative correlation between male and female fertility traits to a non-substantial number (data not shown). It should be also noted that SCR is service sire contribution to pregnancy while HCR is the female contribution to pregnancy. Therefore, SCR is not a direct trait for male fertility, but an indirect male contribution through genetics and potentially epigenetics in sperms [26].

Our phylogenetic analysis indicated that the beef form of *PRDM9* is virtually identical to *B. indicus PRDM9*. Both the *taurus* and *indicus* species descend from the extinct wild aurochs (*Bos primigenius*). However, separate ancient domestication events led to speciation [27] and although these species readily hybridize, male infertility is often observed in the crossbreds [14]. Low levels of haplotype sharing between *B. taurus* and *indicus* breeds have been frequently observed for every analyzed gene because of the recent formation of *B. taurus* × *B. indicus* hybrids in North America [28]. This suggests infiltration into the Holstein herd of the indicine *PRDM9,* which induces unique recombination hotspot regions. These are not compatible with the recombination hotspots mediated by the taurine *PRDM9* and thus drive meiosis in heterozygous individuals toward chromosomal instability and male infertility.

## Conclusions

In Holstein cattle, the breeding scheme for female fertility has been complicated by a negative correlation between this trait and milk production [29]. We show that this scheme is further complicated by the negative genetic correlation between male and female fertility that is encoded in the BTA1 telomere. Cloning the taurine *PRDM9* gene, which is the common form carried by Holstein haplotypes of this region, we demonstrated the infiltration of a rare indicine *PRDM9* variant into the Holstein population. We suggest that during meiosis, in heterozygous males, this may induce incompatibility in the localization of recombination hotspots, destabilize genome integrity, and cause male infertility due to defects in spermiogenesis. However, the indicine *PRDM9* variant was associated with a favorable effect on female fertility, which would explain the survival of this variant and the general negative correlation of *R* = − 0.3 observed between male and female fertility traits in US Holsteins. Further research is needed to explain the mechanism underlying this positive effect on female fertility, and to devise a methodology that will unlink it from the observed negative effect on male fertility.

## Methods

### Deep sequencing and analysis of bovine genomes

The current reference genome is based on the Hereford beef breed. To find variations between the dairy and beef species that may underlie the differences in *PRDM9*, DNA was extracted from thawed frozen semen of a single Holstein sire (JJ, HOLISRM000000007424) and was deep-sequenced using the Illumina HiSeq2000 platform according to the manufacturer’s paired-end protocol. Average fragment length was 580 bp, and 100-bp sequence reads were obtained from both ends. DNA sample was applied to two lanes; yielding ~ 30-fold (906,996,192 reads) coverage for this sample. The reference gene sequence was then used as a template for mapping these DNA-Seq reads using GAP5 software [30]. BWA options for this mapping were set to bam bwasw -t 8 -T 60 [31]. The assembled sequence of this sire gene was submitted under ENA accession nos. ERS3326200 (BAM format) and LR536713 (annotated gene sequence).

Additional genomic sequences of the *PRDM9* locus were reconstructed using DNA-Seq reads located in NCBI’s SRA and the Nucleotide BLAST tool (GenBank accession No. PRJNA277147). The reference gene sequence was then used as a template for mapping these DNA-Seq reads following the above mention procedures for assembling our own data. Further analysis of variation was performed with Sanger sequencing: DNA was amplified using PCR primers (Additional file 1: Table S1) and the Bio-X-ACT™ Long Kit (Bioline Ltd., London, UK) according to the manufacturer’s instructions under the following conditions: 30 cycles for 40 s at 92 °C, 60 s at 63 °C and 60 s at 68 °C. The PCR products were separated on agarose gels, excised, and purified with AccuPrep® Gel Purification Kit (BioNeer Corp., Seoul, Korea). Chromatograms were obtained by ABI3730 sequencing using a BigDye® Terminator v1.1 Cycle Sequencing Kit (Applied Biosystems, Foster City, CA, USA). Detection and characterization of indels were performed using ShiftDetector and the ABI tracefiles [32].

### Cloning of PRDM9 exon10 sequence

*PRDM9* DNA fragments were amplified with subcloning primers (Additional file 1: Table S1) using Hy-Fy High Fidelity Mix (Hy Laboratories Ltd., Rehovot, Israel). The amplified products were digested by restriction enzymes, purified from a 1% agarose gel by Gel/PCR DNA Fragments Kit (Geneaid Biotech Ltd., Taipei, Taiwan) and ligated into the pGEM®-T Easy Vector (Promega, Madison, WI, USA) using *EcoRI* and *NcoI* sites and T4 DNA ligase (Promega). These cloned DNA fragments were subjected to Sanger dideoxy sequencing using primers for SP6 and T7 promoters in pGEM-T Easy and an additional primer within the insert (Additional file 1: Table S1).

### The dataset, haplotype phasing and trait-association analysis

Using Illumina (San Diego, CA, USA) BovineSNP50 BeadChip genotypes, four traits were analyzed: cow, heifer and daughter fertilities (CCR, HCR and DPR, respectively), and sire conception rate (IL-SCR) as previously described [33, 34]. Briefly, IL-SCR was calculated based on a linear model and 5,658,632 insemination records of 1597 sires with a minimum 250 inseminations per sire delivered by a qualified inseminator with a minimum 250 inseminations per year. Fixed effects were insemination number, AI institute, geographical region, and calendar month. Analysis of cows also included the fixed effects of parity, calving status, and day in milk at insemination. Random effects included in the model were herd-year season, insemination technician, sire of cow, and service sire. The standard deviation for IL-SCR evaluations was 0 ± 0.024 and mean reliability was 78.2%. DNA was extracted from the semen of 1750 Holstein bulls used for AI in Israel. The bulls’ identity, relationship and genetic breeding values are available at http://www.icba-israel.com/cgi-bin/bulls/en/bl_main.htm. The dataset of IL sires, including genotyping data and SCR values is available in Excel format (Additional file 2).

Association for BTA1 SNPs was determined using PLINK [16], activating the haplotype sliding-window and bootstrapping options (−-hap-window 10 --hap-linear --mperm 100,000). Haplotype spanning of the *PRDM9* gene, consisting of 10 SNPs within positions 157,229,645–157,542,408 (build ARS-UCD1.2), was chosen for further analyses. For this haplotype, phasing was corroborated using the rule-based Large-Scale Pedigree Haplotyper (LSPH) software [18]. The genetic correlations between traits or between markers’ substitution effects were estimated as Pearson’s correlation coefficients. These coefficients of correlation were calculated using R package [35] or CORREL function in Excel spreadsheet (Microsoft Corporation, Santa Rosa, CA, USA), respectively.

### US Holstein samples and analysis

The data used were part of the 2018 US genomic evaluations from the Council on Dairy Cattle Breeding (CDCB), consisting of 1,953,934 Holstein cattle from the national dairy cattle database. Estimated breeding values (EBVs) of four fertility traits were analyzed: SCR, DPR, HCR and CCR. We only included those animals with both available genotype and trait reliability larger than the parent average. A detailed description of the data is provided in Table 6.

**Table 6:** Description of number of animals, estimated breeding value summary statistics and average of their reliability

Trait	N	Mean ± SD	Min	Max	Reliability
SCR	903,690	2.85 **±** 0.25	1.79	5.01	0.38
DPR	836,623	−14.41 **±** 2.73	−30.39	7.24	0.33
HCR	922,913	−0.62 **±** 2.30	−16.2	11.2	0.28
CCR	794,362	−12.22 **±** 3.42	−32.6	7.34	0.32

The genotype data from different SNP arrays were imputed to a common dataset of 4340 SNPs on BTA1 using FindHap version 3 [36]. Then, nine telomeric SNPs were analyzed: ARS-BFGL-NGS-73542, ARS-BFGL-NGS-19721, ARS-BFGL-NGS-101788, BTA-105868-no-rs, BTB-01585499, ARS-BFGL-NGS-113905, ARS-BFGL-NGS-90894, ARS-BFGL-NGS-83544, and Hapmap26498-BTA-33060. The association studies were performed using PLINK v 1.07 software [16]. Following Garrick et al.*,* 2009 [37], association analysis was also performed using deregressed EBVs (dEBVs) and removing the parent effect from the individual’s EBV. The substitution effects estimated based on dEBVs were highly correlated with those obtained using EBVs (*R* = 0.956, data not shown).

### Analysis of evolutionary relationships

The evolutionary history of the *PRDM9* ZnF-array alleles was inferred using the Neighbor-Joining method. Evolutionary analyses were conducted in MEGA6 [38]. Briefly, the best model was selected according to the lowest Bayesian Information Criterion (BIC) scores. The optimal tree was identified by the bootstrap test (1000 replicates). The evolutionary distances were computed using the JTT matrix-based method in units of number of amino acid substitutions per site. The rate variation among sites was modeled with a gamma distribution (shape parameter = 2.53). All positions containing gaps and missing data were eliminated. There were a total of 342 positions in the final dataset.

## Additional files

Additional file 1: **Table S1.** Primer pairs used for PCR amplification and sequencing of the *PRDM9* gene; and nucleotide sequences of *PRDM9* exon10, partial coding sequences associated with the haplotype and individuals studied. (PDF 362 kb)

Additional file 2: The dataset of IL sires, including genotyping data and SCR values. (XLSX 218 kb)

## Data Availability

Sequence data have been submitted to ENA under accession no. PRJEB31626. The dataset of IL sires, including genotyping data and SCR values, are presented in Additional file 2.

## Abbreviations

AI: Artificial insemination
BIC: Bayesian information criterion
CCR: Cow conception rate
CDS: Coding sequence
DPR: Daughter pregnancy rate
HCR: Heifer conception rate
NGS: Next generation sequencing
SCR: Sire conception rate
