# Supplementary material for: Bos taurus–indicus hybridization correlates with intralocus sexual-conflict effects of PRDM9 on male and female fertility in Holstein cattle
Source: BMC Genet. 2019 Aug 28;20:71. doi: 10.1186/s12863-019-0773-5 (PMC6714232; doi:10.1186/s12863-019-0773-5)
Supplement: Supplementary file 1 — Table S1. Primer pairs used for PCR amplification and sequencing of the PRDM9 gene; and nucleotide sequences of PRDM9 exon10, partial coding sequences associated with the haplotype and individuals studied. (PDF 362 kb) [file 12863_2019_773_MOESM1_ESM.pdf]

Table S1. Primer pairs used for PCR amplification and sequencing of the*PRDM9* gene.

#	Role	Forward primer		Reverse primer	
		**Sequence 5′-> 3′**	**Position**	**Sequence 5′-> 3′**	**Position**
**1**	*Ex10 PCR*	AATCGTGCACTGTCAACACC	Int9	TCCCTGAATTGGAGTCTTGG	Ex10
**2**	*Ex10 sequencing*	AGCGATATTCCGATCCACAC	Ex10		
**3**	*Ex10 Hap9 specific*	TGCATGAGAGAATCACAGAG	Ex10		
**4**	*Ex10 most haplotypes*	TACATGAGAGAATCACAGAA	Ex10		
**5**	*Sub-cloning*	CATGCCATGGAATCGTGCACTGTCAACACC	*NcoI*+Int9	CCGGAATTCTCCCTGAATTGGAGTCTTGG	*EcoRI*+Ex10
**6**	*Vector sequencing*	TAATACGACTCACTATAGGG	T7	TATTTAGGTGACACTATAG	SP6
**7**	*Insert sequencing*	GGCAGGGGTCTCAGGGATT	Ex10		

>*PRDM9* exon10, partial CDS associated with haplotypes #1;#3;6
CAGAATCGAAGCCCAAGATCCACCCATGTGCCTCCTGCTCTCTGGCCTTCTCCAGTCAGAAG
TTCCTCAGCCAACATGTCCAACACAATCACCCCTCTCAGACCCTCCTGAGACCATCTGCAAG
AGACTACCTGCAACCAGAGGATCCCTGCCCAGGCAGTCAAAATCAGCAGCAGCGATATTCCG
ATCCACACAGCCCAAGTGACAAACCTGAGGGTCGTGAGGTCAAGGACAGGCCCCAACCTTTG
CTGAAAAGCATAAGGCTGAAGAGGATTTCAAGGGCCTCCTCCTACTCACCCAGAGGACAAAT
GGGGGCTTCTGGGGTACATGAGAGAATCACAGAAGAGCCCAGCACAAGCCAGAAACCGAATC
CAGAGGACACAGGCAAATTATTCATGGGGGCAGGGGTCTCAGGGATTATAAAAGTCAAGTAC
GGAGAGTGTGGGCAAGGATCCAAGGATCGGTCAAGTCTCATCACAAACCAGAGGACACACAC
AGGGGAGAAGCCCTATGTTTGCGGGGAGTGTGGGCGAAGCTTCAGTCGGAAGGACCATCTCA
TCACACACCAGAGGACACACACAGGGGAGAAGCCCTATGTTTGCGGGGAGTGTGGGCGAAGC
TTCAGTCAGAAGTCCACTCTCATCACACACCAGAGGACACACACAGGGGAGAAGCCCTATGT
TTGCGGGGAGTGTGGGCAAAGCTTCAGTCAGAAGGGCCGTCTCATCACACACCAGAGGACAC
ACACAGGGGAGAAGCCCTATGTTTGCGGGGAGTGTGGGCGAAGCTTCAGTCAGAAGTCCACT
CTCATCACACACCAGAGGACACACACAGGGGAGAAGCCCTATGTTTGCGGGGAGTGTGGGCG
AAGCTTCAGTCAGAAGTCCACTCTCATCACACACCAGAGGACACACACAGGGGAGAAGCCCT
ATGTTTGCGGGGAGTGTGGGCAAAGCTTCAGTCAGAAGGGCTATCTCATCACACACCAGAGG
ACCCACAGGGGAGAAGCCTTATGTTGCAGGGAGTGTGAGTGA
>*PRDM9* exon10, partial CDS associated with haplotypes #2;#15
CAGAATCGAAGCCCAAGATCCACCCATGTGCCTCCTGCTCTCTGGCCTTCTCCAGTCAGAAG
TTCCTCAGCCAACATGTCCAACACAATCACCCCTCTCAGACCCTCCTGAGACCATCTGCAAG
AGACTACCTGCAACCAGAGGATCCCTGCCCAGGCAGTCAAAATCAGCAGCAGCGATATTCCG
ATCCACACAGCCCAAGTGACAAACCTGAGGGTCGTGAGGTCAAGGACAGGCCCCAACCTTTG
CTGAAAAGCATAAGGCTGAAGAGGATTTCAAGGGCCTCCTCCTACTCACCCAGAGGACAAAT
GGGGGCTTCTGGGGTACATGAGAGAATCACAGAAGAGCCCAGCACAAGCCAGAAACCGAATC
CAGAGGACACAGGCAAATTATTCATGGGGGCAGGGGTCTCAGGGATTATAAAAGTCAAGTAC
GGAGAGTGTGGGCAAGGATCCAAGGATCGGTCAAGTCTCATCACAAACCAGAGGACACACAC
AGGGGAGAAGCCCTATGTTTGCGGGGAGTGTGGGCGAAGCTTCAGTCGGAAGGACCATCTCA
TCACACACCAGAGGACACACACAGGGGAGAAGCCCTATGTTTGCGGGGAGTGTGGGCGAAGC
TTCAGTCAGAAGTCCACTCTCATCACACACCAGAGGACACACACAGGGGAGAAGCCCTATGT
TTGCGGGGAGTGTGGGCGAAGCTTCAGTCAGAAGGGCCGTCTCATCACACACCAGAGGACAC
ACACAGGGGAGAAGCCCTATGTTTGCGGGGAGTGTGGGCGAAGCTTCAGTCAGAAGTCCACT
CTCATCACACACCAGAGGACACACACAGGGGAGAAGCCCTATGTTTGCGGGGAGTGTGGGCG
AAGCTTCAGTCAGAAGTCCACTCTCATCACACACCAGAGGACACACACAGGGGAGAAGCCCT
ATGTTTGCGGGGAGTGTGGGCAAAGCTTCAGTCAGAAGGGCTATCTCATCACACACCAGAGG
ACCCACAGGGGAGAAGCCTTATGTTGCAGGGAGTGTGAGTGA
>*PRDM9* exon10, partial CDS associated with haplotypes #4
CAGAATCGAAGCCCAAGATCCACCCATGTGCCTCCTGCTCTCTGGCCTTCTCCAGTCAGAAG
TTCCTCAGCCAACATGTCCAACACAATCACCCCTCTCAGACCCTCCTGAGACCATCTGCAAG
AGACTACCTGCAACCAGAGGATCCCTGCCCAGGCAGTCAAAATCAGCAGCAGCGATATTCCG
ATCCACACAGCCCAAGTGACAAACCTGAGGGTCGTGAGGTCAAGGACAGGCCCCAACCTTTG
CTGAAAAGCATAAGGCTGAAGAGGATTTCAAGGGCCTCCTCCTACTCACCCAGAGGACAAAT
GGGGGCTTCTGGGGTACATGAGAGAATCACAGAAGAGCCCAGCACAAGCCAGAAACCGAATC
CAGAGGACACAGGCAAATTATTCATGGGGGCAGGGGTCTCAGGGATTATAAAAGTCAAGTAC
GGAGAGTGTGGGCAAGGATCCAAGGATCGGTCAAGTCTCATCACAAACCAGAGGACACACAC
AGGGGAGAAGCCCTATGTTTGCGGGGAGTGTGGGCGAAGCTTCAGTCGGAAGGACCATCTCA
TCACACACCAGAGGACACACACAGGGGAGAAGCCCTATGTTTGCGGGGAGTGTGGGCGAAGC
TTCAGTCAGAAGTCCACTCTCATCACACACCAGAGGACACACACAGGGGAGAAGCCCTATGT
TTGCGGGGAGTGTGGGCAAAGCTTCAGTCAGAAGGGCCGTCTCATCACACACCAGAGGACAC
ACACAGGGGAGAAGCCCTATGTTTGCGGGGAGTGTGGGCGAAGCTTCAGTCAGAAGTCCACT
CTCATCACACACCAGAGGACACACACAGGGGAGAAGCCCTATGTTTGCGGGGAGTGTGGGCG
AAGCTTCAGTCAGAAGTCCACTCTCATCACACACCAGAGGACACACACAGGGGAGAAGCCCT
ATGTTTGCGGGGAGTGTGGGCGAAGCTTCAGTCAGAAGGGCTATCTCATCACACACCAGAGG
ACCCACAGGGGAGAAGCCTTATGTTGCAGGGAGTGTGAGTGA
>*PRDM9* exon10, partial CDS associated with haplotypes #5;7

CAGAATCGAAGCCCAAGATCCACCCATGTGCCTCCTGCTCTCTGGCCTTCTCCAGTCAGAAG
TTCCTCAGCCAACATGTCCAACACAATCACCCCTCTCAGACCCTCCTGAGACCATCTGCAAG
AGACTACCTGCAACCAGAGGATCCCTGCCCAGGCAGTCAAAATCAGCAGCAGCGATATTCCG
ATCCACACAGCCCAAGTGACAAACCTGAGGGTCGTGAGGTCAAGGACAGGCCCCAACCTTTG
CTGAAAAGCATAAGGCTGAAGAGGATTTCAAGGGCCTCCTCCTACTCACCCAGAGGACAAAT
GGGGGCTTCTGGGGTACATGAGAGAATCACAGAAGAGCCCAGCACAAGCCAGAAACCGAATC
CAGAGGACACAGGCAAATTATTCATGGGGGCAGGGGTCTCAGGGATTATAAAAGTCAAGTAC
GGAGAGTGTGGGCAAGGATCCAAGGATCGGTCAAGTCTCATCACAAACCAGAGGACACACAC
AGGGGAGAAGCCCTATGTTTGCGGGGAGTGTGGGCGAAGCTTCAGTCGGAAGGACCATCTCA
TCACACACCAGAGGACACACACAGGGGAGAAGCCCTATGTTTGCGGGGAGTGTGGGCGAAGC
TTCAGTCAGAAGTCCACTCTCATCACACACCAGAGGACACACACAGGGGAGAAGCCCTATGT
TTGCGGGGAGTGTGGGCAAAGCTTCAGTCAGAAGGGCCGTCTCATCACACACCAGAGGACAC
ACACAGGGGAGAAGCCCTATGTTTGCGGGGAGTGTGGGCGAAGCTTCAGTCAGAAGTCCACT
CTCATCACACACCAGAGGACACACACAGGGGAGAAGCCCTATGTTTGCGGGGAGTGTGGGCG
AAGCTTCAGTCAGAAGTCCACTCTCATCACACACCAGAGGACACACACAGGGGAGAAGCCCT
ATGTTTGCGGGGAGTGTGGGCGAAGCTTCAGTCAGAAGTCCACTCTCATCACACACCAGAGG
ACACACACAGGGGAGAAGCCCTATGTTTGCGGGGAGTGTGGGCAAAGCTTCAGTCAGAAGGG
CTATCTCATCACACACCAGAGGACCCACAGGGGAGAAGCCTTATGTTGCAGGGAGTGTGAGT
GA
>*PRDM9* exon10, partial CDS associated with haplotypes #9;#10;and of DOMINETTE
CAGAATCGAAGCCCAAGATCCACCCATGTGCCTCCTGCTCTCTGGCCTTCTCCAGTCAGAAG
TTCCTCAGCCAACATGTCCAACACAATCACCCCTCTCAGACCCTCCTGAGACCATCTGCAAG
AGACTACCTGCAACCAGAGGATCCCTGCCCAGGCAGTCAAAATCAGCAGCAGCGATATTCCG
ATCCACACAGCCCAAGTGACAAACCTGAGGGTCGTGAGGTCAAGGACAGGCCCCAACCTTTG
CTGAAAAGCATAAGGCTGAAGAGGATTTCAAGGGCCTCCTCCTACTCACCCAGAGGACAAAT
GGGGGTTTCTGGGGTGCATGAGAGAATCACAGAGGAGCCCAGCACAAGCCAGAAACCAAATC
CAGAGGACACAGGCAAATTATTCATGGGGGCAGGGGTCTCAGGGATTATAAAAGTCAAGTAC
GGAGAGTGTGGGCAAGGATCCAAGGATAGGTCAAGTCTCATCACAAACCAGAGGACACACAC
AGGGGAGAAGCCCTATGTTTGCGGGGAGTGTGGGCAAAGCTTCAATCAGAAGTCCACTCTCA
TCACACACCAGAGGACACACACAGGGGAGAAGCCCTATGTTTGCGGGGAGTGTGGGCGAAGC
TTCAATCAGAAGTCCACTCTCATCACACACCAGAGGACACACACAGGGGAGAAGCCCTATGT
TTGCGGGGAGTGTGGGCGAAGCTTCAGTCAGAAGTCCACTCTCATCAAACACCAGAGGACAC
ACACAGGGGAGAAGCCCTATGTTTGCGGGGAGTGTGGGCAAAGCTTCAATCAGAAGTCCACT
CTCATCACACACCAGAGGACACACACAGGGGAGAAGCCCTATGTTTGCGGGGAGTGTGGGCA
AAGCTTCAATCAGAAGTCCACTCTCATCACACACCAGAGGACACACACAGGGGAGAAGCCCT
ATGTTTGCGGGGAGTGTGGGCGAAGCTTCAGTCGGAAGTCCACTCTCATCACACACCAGAGG
ACACACAGAGGAGAAGCCTTATGTTTGCAGGGAGTGTGA
>*PRDM9* exon10, partial CDS associated with a haplotype of JJ
CAGAATCGAAGCCCAAGATCCACCCATGTGCCTCCTGCTCTCTGGCCTTCTCCAGTCAGAAG
TTCCTCAGCCAACATGTCCAACACAATCACCCCTCTCAGACCCTCCTGAGACCATCTGCAAG
AGACTACCTGCAACCAGAGGATCCCTGCCCAGGCAGTCAAAATCAGCAGCAGCGATATTCCG
ATCCACACAGCCCAAGTGACAAACCTGAGGGTCGTGAGGTCAAGGACAGGCCCCAACCTTTG
CTGAAAAGCATAAGGCTGAAGAGGATTTCAAGGGCCTCCTCCTACTCACCCAGAGGACAAGT
GGGGGGGTCTGGGGTGCATGAGAGAATCACAGAAGAGCCCAGCACAAGCCAGAAACTGAATC
CAGGGGACACAGGCAAATTATTCATGGGGGCAGGGGTCTCAGGGATTATAAAAGTCAAGTAC
GGAGAGTGTGGGCAAGGATCCAAGGATCGGTCAAGTCTCATCACAAACCAGAGGACACACAC
AGGGGAGAAGCCCTATGTTTGCGGGGAGTGTGGGCGAAGCTTCAGTCGGAAGGACCATCTCA
TCACACACCAGAGGACACACACAGGGGAGAAGCCCTATGTTTGCGGGGAGTGTGGGCGAAGC
TTCAGTCAGAAGTCCACTCTCATCACACACCAGAGGACACACACAGGGGAGAAGCCCTATGT
TTGCGGGGAGTGTGGGCGAAGCTTCAGTCAGAAGTCCACTCTCATCACACACCAGAGGACAC
ACACAGGGGAGAAGCCCTATGTTTGCGGGGAGTGTGGGCAAAGCTTCAGTCAGAAGGGCCGT
CTCATCACACACCAGAGGACACACACAGGGGAGAAGCCCTATGTTTGCGGGGAGTGTGGGCG
AAGCTTCAGTCAGAAGTCCACTCTCATCACACACCAGAGGACACACACAGGGGAGAAGCCCT
ATGTTTGCGGGGAGTGTGGGCAAAGCTTCAGTCAGAAGGGCTATCTCATCACACACCAGAGG
ACCCACAGGGGAGAAGCCTTATGTTGCAGGGAGTGTGAGTGA

>*PRDM9* exon10, partial CDS associated with haplotype indicus_A
CAGAATCGAAGCCCAAGATCCACCCATGTGCCTCCTGCTCTCTGGCCTTCTCCAGTCAGAAG
TTCCTCAGCCAACATGTCCAACACAATCACCCCTCTCAGACCCTCCTGAGACCATCTGCAAG
AGACTACCTGCAACCAGAGGATCCCTGCCCAGGCAATCAAAATCAGCAGCAGCGATATTCCG
ATCCACACAGCCCAAGTGACAAACCTGAGGGTCGTGAGGTCAAGGACAGGCCCCAACCTTTG
CTGAAAAGCATAAAGCTGAAGAGGATTTCAAGGGCCTCCTCCTACTCACCCAGAGGACAAAT
GGGGGCTTCTGGGGTACATGAGAGAATCACAGAAGAGCCCAGCACAAGCCAGAAACTGAATC
CAGAGGACACAGGCAAATTATTCATGGGGGCAGGGGTCTCAGGGATTATAAAAGTCAAGTAC
GGAGAGTGTGGGCAAGGATCCAAGGATCGGTCAAGTCTCATCACAAACCAGAGGACACACAC
AGGGGAGAAGCCCTATGTTTGCGGGGAGTGTGGGCAAAGCTTCAATCAGAAGTCCACTCTCA
TCACACACCAGAGGACACACACAGGGGAGAAGCCCTATGTTTGCGGGGAGTGTGGGCGAAGC
TTCAATCAGAAGTCCACTCTCATCACACACCAGAGGACACACACAGGGGAGAAGCCCTATGT
TTGCGGGGAGTGTGGGCGAAGCTTCAGTCAGAAGTCCACTCTCATCAAACACCAGAGGACAC
ACACAGGGGAGAAGCCCTATGTTTGCGGGGAGTGTGGGCAAAGCTTCAATCAGAAGTCCACT
CTCATCACACACCAGAGGACACACACAGGGGAGAAGCCCTATGTTTGCGGGGAGTGTGGGCA
AAGCTTCAATCAGAAGTCCACTCTCATCACACACCAGAGGACACACACAGGGGAGAAGCCCT
ATGTTTGCGGGGAGTGTGGGCGAAGCTTCAGTCGGAAGTCCACTCTCATCACACACCAGAGG
ACACACAGAGGAGAAGCCTTATGTTTGCAGGGAGTGTGA
>*PRDM9* exon10, partial CDS associated with haplotype indicus_B
CAGAATCGAAGCCCAAGATCCACCCATGTGCCTCCTGCTCTCTGGCCTTCTCCAGTCAGAAG
TTCCTCAGCCAACATGTCCAACACAATCACCCCTCTCAGACCCTCCTGAGACCATCTGCAAG
AGACTACCTGCAACCAGAGGATCCCTGCCCAGGCAGTCAAAATCAGCAGCAGCGATATTCCG
ATCCACACAGCCCAAGTGACAAACCTGAGGGTCGTGAGGTCAAGGACAGGCCCCAACCTTTG
CTGAAAAGCATAAGGCTGAAGAGGATTTCAAGGGCCTCCTCCTACTCACCCAGAGGACAAAT
GGGGGTTTCTGGGGTACATGAGAGAATCACAGAAGAGCCCAGCACAAGCCAGAAACCAAATC
CAGAGGACACAGGCAAATTATTCATGGGGGCAGGGGTCTCAGGGATTATAAAAGTCAAGTAC
GGAGAGTGTGGGCAAGGATCCAAGGATAGGTCAAGTCTCATCACAAACCAGAGGACACACAC
AGGGGAGAAGCCCTATGTTTGCGGGGAGTGTGGGCAAAGCTTCAATCAGAAGTCCACTCTCA
TCACACACCAGAGGACACACACAGGGGAGAAGCCCTATGTTTGCGGGGAGTGTGGGCGAAGC
TTCAATCAGAAGTCCACTCTCATCACACACCAGAGGACACACACAGGGGAGAAGCCCTATGT
TTGCGGGGAGTGTGGGCGAAGCTTCAGTCAGAAGTCCACTCTCATCAAACACCAGAGGACAC
ACACAGGGGAGAAGCCCTATGTTTGCGGGGAGTGTGGGCAAAGCTTCAATCAGAAGTCCACT
CTCATCACACACCAGAGGACACACACAGGGGAGAAGCCCTATGTTTGCGGGGAGTGTGGGCA
AAGCTTCAATCAGAAGTCCACTCTCATCACACACCAGAGGACACACACAGGGGAGAAGCCCT
ATGTTTGCGGGGAGTGTGGGCGAAGCTTCAGTCGGAAGTCCACTCTCATCACACACCAGAGG
ACACACAGAGGAGAAGCCTTATGTTTGCAGGGAGTGTGA
